# A hospital-wide outbreak of IMI-17-producing *Enterobacter ludwigii* in an Israeli hospital

**DOI:** 10.1186/s13756-021-01036-0

**Published:** 2021-11-29

**Authors:** Vered Schechner, Katya Levytskyi, Ohad Shalom, Alexander Yalek, Amos Adler

**Affiliations:** 1 Section of Epidemiology and Preventative Medicine, Tel-Aviv Sourasky Medical Center, Tel-Aviv, Israel; 2 Sackler Faculty of Medicine, Tel-Aviv University, 6 Weizmann Street, 6423906 Tel-Aviv, Israel; 3 Clinical Microbiology Laboratory, Tel-Aviv Sourasky Medical Center, Tel-Aviv, Israel; 4 Department of Pediatrics, Tel-Aviv Sourasky Medical Center, Tel-Aviv, Israel

**Keywords:** IMI, Outbreak, Carbapenemase

## Abstract

**Background:**

To describe the course and intervention of an hospital-wide IMI-Producing *Enterobacter ludwigii* outbreak.

**Methods:**

This was an outbreak interventional study, done at a tertiary care center in Tel-Aviv, Israel. Data was collected on the course of the outbreak and the demographic and clinical characteristics of all patients involved in the outbreak. The intervention measures included patients’ cohorting, contact isolation precautions, environmental cleaning and screening of contacts. The molecular features and phylogeny of outbreak-related isolates were studied by whole-genome based analysis.

**Results:**

The outbreak included 34 patients that were colonized by IMI-Producing *E. ludwigii* and were identified in 24 wards throughout the hospital. Colonization was identified in the first 72 h of admission in 13/34 patients (38.2%). Most patients (91.2%) were admitted from home and had relatively low level of comorbidities. The majority of them (88%) had no recent use of invasive catheters and none had previous carriage of other multi-drug resistant bacteria. All available isolates harbored the *bla*_IMI-17_ allele and belonged to Sequence-Type 385. With the exception of two isolates, all isolates were closely related with less than a 20-SNP difference between them.

**Conclusions:**

This outbreak had most likely originated in the community and subsequently disseminated inside our institution. More studies are required in order to elucidate the epidemiology of IMI-Producing *E. ludwigii* and the possible role of environmental sources in its dissemination.

## Introduction

Since the beginning of the millennium, carbapenemase-producing Enterobacterales (CPE) have become a major problem in health-care systems worldwide. In Israel, a nationwide outbreak of CPE emerged in 2006, consisting primarily of nosocomial spread of KPC-producing *Klebsiella pneumoniae* [1]. Even prior to that outbreak, the first reported cases of CPE in Israel were in fact due to KPC-producing *Enterobacter cloacae* [2]*.* In addition to KPC, several studies described outbreaks that were caused by VIM-producing *E. cloacae* [3, 4]*.* In other countries, carbapenemase-producing *E. cloacae* is the third most common CPE species (following *K. pneumoniae* and *Escherichia coli*) [5] or even becoming as prevalent as CPE *K. pneumoniae* [6].

In addition to common carbapenemase enzymes such as KPC or VIM that can be found in many different Enterobacterales species, the *E. cloacae* complex (ECC) species may produce a species-unique carbapenemase named IMI. This type-A enzyme is mostly located on the bacterial chromosome, and has been reported, albeit rarely, from around the globe [7]. In Israel, testing for IMI by clinical laboratories is not routinely mandated [8] but this carbapenemase was still reported from 2.7% of all CPE in 2019 in Israel [9]. In June 2019 we have encountered an institution-wide outbreak of 34 cases of IMI-producing ECC that lasted for four months. The goals of this study were to describe this outbreak and to postulate regarding its origin and its clinical-epidemiological significance.

## Methods

### Setup and settings

The Tel-Aviv Sourasky Medical Center (TASMC) is a 1,400-bed, tertiary care center in Tel-Aviv, Israel. The policies for the selection of patients for CPE screening by rectal surveillance cultures were determined according to instructions of the Israeli Ministry of Health [8]. Briefly, surveillance cultures are collected in patients following either 1) a recent contact with another CPE carrier (post-contact surveillance); 2) in patients admitted from suspected endemic environment, such as those admitted from other institutions (admission surveillance) and 3) periodically in specific high-risk wards (e.g., ICU, hematology-oncology). All positive samples of CPE in TASMC are reported on real time to the hospital infection prevention and control unit. CPE carriers are placed in specific wards (i.e., CPE cohorts) with dedicated nursing staff and strict contact isolation precautions.

### Patients and data collection

All patients with a positive sample of IMI-producing ECC between 01/01/2018 and 30/09/2019 were included. Data was collected from the computerized patient’s files and included: 1) demographic data; 2) previous use of healthcare services or medications; 3) baseline medical conditions, including the Activities of Daily Living (ADL) score and the Charlson Clinical Score (CCS). The study was approved by the institutional Ethics Committee. The isolation of isolate following 72 h of admission was defined as 'hospital acquisition' and 'healthcare exposure' was defined as either inpatient admission with 6 month or Surgery within 30 days.

### Detection of carbapenemase and microbiological methods

Surveillance for CPE colonization was done by rectal swabs. Swabs were inoculated onto the CHROMAgar™ and mSuperCARBA™ media (produced under license by a local manufacturer, HyLabs, Rehovot, Israel) and incubated for 16–18 h at 37ºC. The analysis of suspicious colonies grown on the media was done at the Laboratory of Molecular Epidemiology and Antimicrobial Resistance according to instructions of the Israeli Ministry of Health [8] that included PCR assays for the following genes- *bla*_KPC_, *bla*_NDM_, *bla*_OXA-48_ and *bla*_VIM_ using an in-house assays [10]. PCR for *bla*_IMI_ was also performed routinely on all suspicious colonies using the following primers: IMIF-CCATATCACCTAATGACATTCC; IMIR-GCAAATGAACGATTTCCATTATGTA. In addition, carbapenemase activity was tested by the the β-CARBA test (Bio-Rad, Marnes-la-Coquette, France). Species determination was done by the VITEK-MS® MALDI-ToF system (bioMerieux, Marcy l'Etoile, France). Antimicrobial susceptibility testing was done by the VITEK-2 system (bioMerieux, Marcy l'Etoile, France).

### Whole-genome sequencing and molecular analysis

Whole genome sequencing (WGS) was done using the Illumina MiSeq system. Libraries were prepared using Nextera DNA Flex Library Prep Kit (Illumina, 20018705) along with Nextera DNA UD Indexes (Illumina, 20027213) were used to tagment the DNA libraries for sequencing. The library was normalized to 4nMol and sequenced on the Illumina MiSeq platform, using Illumina MiSeq reagent kit v2 300 cycles (2X150 bp), according to the manufacturer’s instructions. After sequencing of each library, FASTAQ files were imported into CLC Genomics Workbench version 12.0.3 (Qiagen, Denmark) included raw reads firstly trimmed based on read quality and to remove adapters, and the reads were de novo assembled into contigs. Illumina reads were mapped to a reference genome (*E. cloacae* EcWSU1 NC_016514) and annotated using the Microbial Genomic Module of the CLC Workbench software. Molecular species identification was done by comparison of the *dnaJ* sequences using the BIBI database [11]. Phylogenetic analysis was done first multi-locus sequence typing (MLST) by comparing the WGS-generated sequences to the MLST database (https://pubmlst.org/ecloacae/) [12] followed by SNP calling for sequence-type (ST)-385 isolates. SNP calling was done using the CLC Workbench software [13, 14] of all shared SNPs with a coverage of ≥ 10, for a total input of 48,969 SNPs. Phylogenetic tree was constructed using the NJ algorithm and included the metadata of isolation date, timing of carriage identification (before/after 3 days of admission) and admitting department. The *bla*_IMI_ allele was determined by WGS.

## Results

### Initial detection of the outbreak

From January 2018 until June 2019, only a single IMI-El carrier was detected (06.30.2018). The outbreak was noticed following the detection of seven IMI-producing *E. ludwigii* (IMI-El) carriers between 6–9 of June 2019. Two patients were from the same ward, and were screened as following the detection of a positive KPC-producing *K. pneumoniae* case in the same ward. The other five patients were from different wards, and were detected incidentally on routine screening.

### Outbreak investigation and intervention

The abrupt onset of the isolation of IMI-El in unrelated patients had led to the assumption of a pseudo-outbreak, possibly due to laboratory contamination. Therefore, patients were not transferred to the CPE cohort ward and were kept under contact isolation.

Repeat testing of the original isolates using new reagents confirmed the initial identification of IMI-El in all samples, as was repeat culturing of several positive swabs. Repeat sampling of the seven initial IMI-El carriers was first negative, but one carrier was later tested positive. All of the laboratory bio-safety cabinets were disinfected using H_2_O_2_ gas and the lab was thoroughly cleansed. However, as additional new IMI-El carriers continued to be identified the outbreak was deemed to be real.

**Fig. 1:**
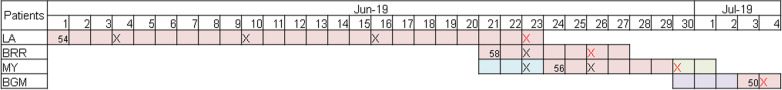
Epidemic curve of IMI-producing *E. ludwigii* (IMI-El) in Surgical Unit C. The curve describes cases detected during or following hospitalization in the surgical unit C (1 June–4 July). Colors represent specific wards; surgical unit C (pink), surgical step-up unit (blue), surgical unit B (purple), geriatric unit (green). Numbers represent the room numbers in surgical unit C. X = a negative rectal test for carbapenemase-producing Enterobacterales. X = a positive rectal test for IMI-El

Overall, 17 new cases were detected in June, eight in July and in August and one in September 2019 (total- 34 cases). All IMI-El cases were detected in surveillance culture without clinical infections, and were identified in 24 different wards with small clusters of 2–3 patients per ward. The largest cluster occurred in Surgical Unit C, where four patients were identified (Fig. 1). IMI-El carriers were managed in a similar manner to other types of CPE, including cohorting, contact precautions, environmental cleaning and screening of contacts. Subsequently, only one IMI-El case was identified in September 2019 but isolated cases (once per month or less) continued to be identified ever since.

### Demographic and epidemiological features of IMI-producing E. ludwigii carriers

The IMI-El carriers population had a mean age of 67 years (range- 27–95 years) and 17/34 (50%) were females. The majority of patients were admitted from home (91.2%), mostly via the Emergency Department (79.4%) to different medical and surgical wards (Table 1). IMI-El was identified in the first 72 h of admission in 13/34 patients (38.2%), of which twelve were admitted from home. Most patients had some degree of exposure to healthcare facilities within the preceding six months (70.5%) including surgery within 30 days in twelve (35.3%) patients, but only few were receiving immunosuppression therapy and none had hematologic malignancy. Likewise, most patients had relatively low CCS and high ADL score upon admission and only one had previous multi-drug resistant bacteria (MDR) infection/colonization. Beside the four ICU patients, none had recent use of invasive devices but recent use of antimicrobials, especially β-lactams (35.2%) was common.

**Table 1:** Demographic and epidemiological features of IMI-producing *E. ludwigii* carriers

Variable, n (%)		
Age (years), mean (SD), range		67 (18.4), 27–95
Female gender		17 (50)
Admission	Home residence	31 (91.2)
	Non-elective	27 (79.4)
	Admitting service	Internal medicine-13 (38), General Surgery-7 (20.5), other surgical services-7 (20.5), ICU-4 (11.7), other-3 (8.8)
IMI-producing *E. ludwigii* isolation from admission (days)- range, no. within 3 days (%)		0–76, 13 (38.2)
Healthcare exposure	Healthcare admission with 6 month	24 (70.5)
	Surgery within 30 days	12 (35.3)
Immunosuppression within 30 days	Chemotherapy	1 (2.9)
	Systemic steroids	3 (8.8)
	Cancer	10 (29.4)
Comorbidities scores, mean (SD), median	ADL^1^	83.2 (25.3), 100
	Charlson	4.24 (3.2), 4
Invasive device within 1 week	Urine catheter	4 (11.7)
	Central venous catheter	0
	Mechanical ventilation	4 (11.7)
Antimicrobial use within 30 days	Carbapenem	2 (5.9)
	Other β-lactams	12 (35.2)
	Aminoglycosides	3 (8.8)
	Quinolones	1 (2.9)
	Macrolides	1 (2.9)
	Vancomycin	3 (8.6)
	Other	4 (11.7)

^1^ADL, activities of daily living

### Microbiological and molecular characteristics of IMI-producing E. ludwigii isolates

The analysis included 27 isolates that were available out of the 34 outbreak’s cases. We also included five unrelated IMI-producing ECC isolated that were previously identified in TASMC. The outbreak-related isolates were all identified as *E. ludwigii* (a member of the ECC) with an average nucleotide identity (ANI) of 99.9% followed by *E. cloacae* with an ANI of 96%. The isolates were resistant to ceftriaxone, piperacillin-tazobactam, ertapenem and meropenem but were tested negative for carbapenemase activity by the β-CARBA test. The isolates were susceptible to ceftazidime, ciprofloxacin, amikacin, gentamicin, nitrofurantoin and trimethoprim-sulfamethoxazole. The isolates harbored the *bla*_IMI-17_ allele and belonged to ST-385. The five unrelated isolates were all identified as *E. cloacae* with an ANI of 100% followed by *E. ludwigii* with an ANI of 95.8%. The isolates harbored the *bla*_IMI-12_ allele (four isolates) or the *bla*_IMI-16_ allele (one isolate). Two isolates belonged to the newly assigned ST-1335 and the rest were of unrelated ST’s (ST-731, 1253 and 1334).

**Fig. 2:**
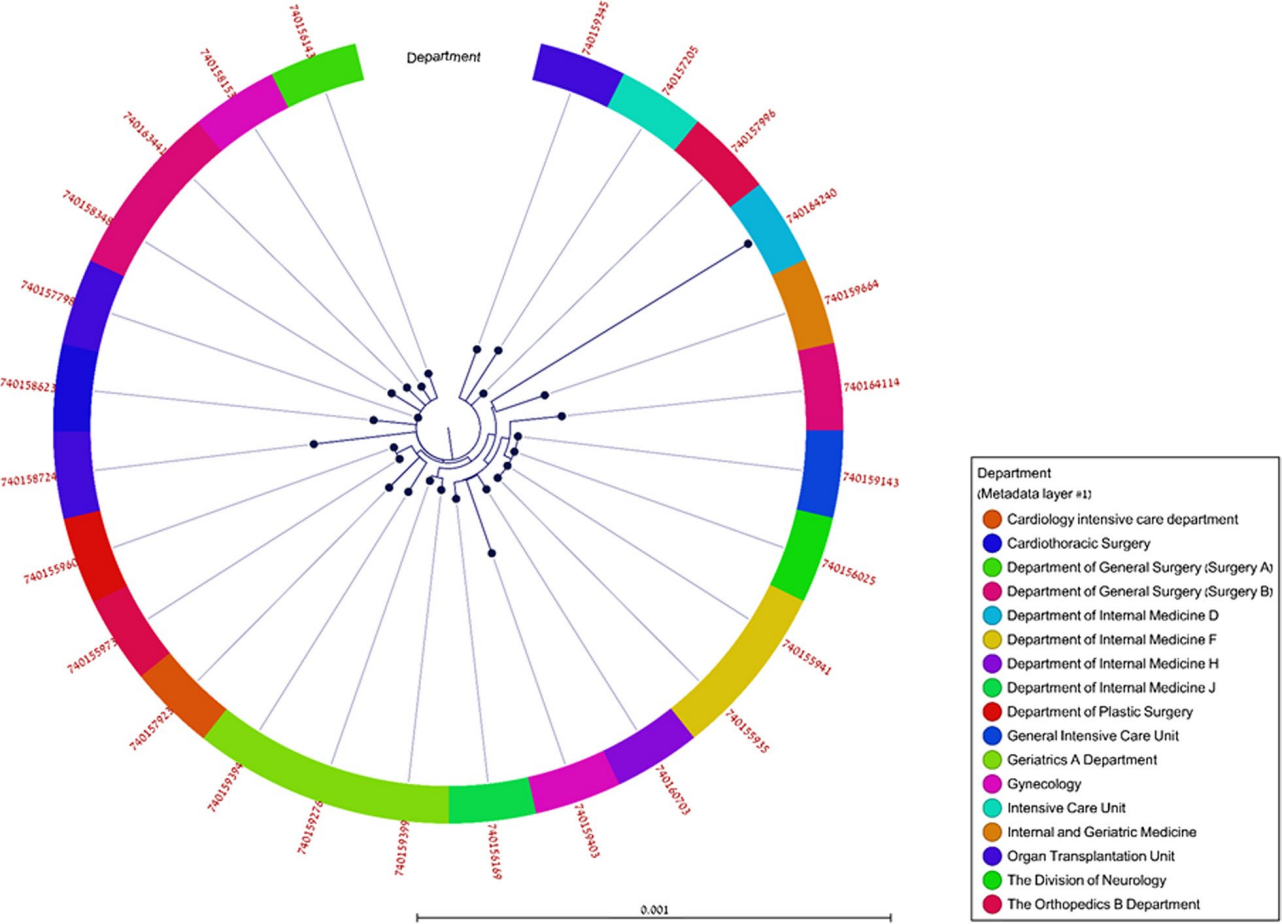
SNP-based phylogenetic analysis of the 27 outbreak-related ST-385 IMI-producing *E. ludwigii* isolates. Metadata layer include the admitting department

The results of the phylogenetic analysis of the ST-385 outbreak-related isolates is presented in Fig. 2. With the exception of two isolates, all 25 isolates were closely related with less than a 20-SNP difference between them. Overall, there was little convergence in relation to date (data not shown) and location of the isolation. Isolates 740158724 and 740164240 maximal differences from the other 25 isolates were 24 and 44 SNP’s, respectively. Isolate 740164240 was the only one isolated from Internal Medicine D ward.

## Discussion

Very little is known about the epidemiology of IMI-producing ECC. Most published studies are mainly isolated case reports [15, 16] or molecular studies [17], that does not include any clinical or epidemiological data. Interestingly, almost all of these studies were reported from East Asia. Thus, our study provides novel data regarding the clinical-epidemiological features of IMI-ECC.

An integral part of outbreak investigation is the detection of its initial source, whether inside or outside the healthcare facility. Typically, the origin is deemed as community-acquired when the infection is detected within 48–72 h of admission. Such distinction is more problematic in case of colonization only, since it is determined by the timing of testing rather than the onset of clinical symptoms. The source of the outbreak described in this article is puzzling from many perspectives. Although colonization was detected in most of the patients after more than three days, suggesting hospital-related acquisition, in substantial number of patients (13, 38.2%) it was detected prior to that, including during the first day of admission. Second, the cases were identified throughout the hospital with no connecting link between most of them. Third, detection of carriage was transient in many of the patients. Fourth, similar outbreaks were not reported from outside of our institution during that time period. Lastly, although the molecular analysis had confirmed that all isolates belonged to a single sequence type of *E. ludwigii*, we were not able to discern the origin of the outbreak or to track its dissemination via the WGS-based analysis. However, the high level of similarity (< 20 SNP’s difference) that was observed between most of the strains suggest that the outbreak originated from a single source and had disseminated within a short time frame.

What can therefore be the epidemiological explanation for this outbreak? Our initial assumption was that laboratory contamination was responsible, at least in part to a pseudo-outbreak. This hypothesis was disproved for the most part by the lack of evidence for environmental source in the lab and the continued appearance of new cases even after thorough laboratory decontamination. Since neither community or healthcare acquisition alone can account for this outbreak, the explanation is probably a combination of both. Of note, community sources of IMI-producing ECC were identified in one report from Myanmar [18] and was suspected in additional reports from two islands in the Indian ocean [14, 19].

Additional features that support the hypothesis that community acquisition of IMI-El played a part in this outbreak are the demographic and clinical characteristics of the carriers. The majority of the patients in our study were admitted from home and had relatively low level of comorbidity and high level of functioning. The majority of them had no recent use of invasive catheters and none had previous carriage of other MDR bacteria. This is in contrast with previous reports of patients infected/colonized with KPC- or VIM- producing Enterobacter species [2, 3], where most patients were either critically ill or had considerable other comorbidities. Moreover, none of the patients in our study had clinical infection caused by IMI-El, as was also reported in another report of IMI-producing ECC outbreak [14]. The seemingly low level of morbidity associated with IMI-producing ECC, raises the question whether IMI-producing ECC should be monitored and managed with the same vigilance as other CPE. In addition to the Infection Control implications, this question merits important microbiological considerations.

IMI-producing ECC poses unique challenges to microbiological laboratories. First, it is not detected by certain selective media as well as other, more common CPE types [20]. Second, it reacts poorly in both in-house and commercial carbapenem-hydrolysis assays [21], as was also observed in our study. Third, with the exception of one PCR assay [22], IMI is absent from all the commercial assays designed to detect carbapenemase, either by PCR or by the novel lateral-flow assays. Fourth, the routine detection of IMI is not requested by professional or national microbiological guidelines (including the Israeli [8]) and thus decreases the laboratory awareness for this type of CPE. Together, these factors hampers the detection of IMI-producing ECC and consequently, one can assume that its prevalence is underestimated compared with other CPE types. Thus, its detection in 2% of all new CPE cases in Israel in 2019 (compared with VIM in 5%) [9] is surprisingly high considering the difficulties listed above.

When and how should IMI-producing ECC be sought for, considering the aforementioned methodological challenges? Although this type of CPE is considered rare, our experience demonstrates the potential danger of rapid dissemination of this CPE. Had we lacked the ability to identify it in real time, it’ll have probably spread to the most susceptible populations in our hospital where the risk of clinical infection and mortality would have been much higher [23]. Hence, IMI-producing ECC should be suspected whenever meropenem-resistant ECC is identified and the isolates is tested negative by PCR to the more common CPE genes (i.e., *bla*_KPC_, *bla*_NDM_, *bla*_OXA-48_ and *bla*_VIM_). Such isolate should be tested by *bla*_IMI_-PCR either locally or by a reference laboratory, irrespective of the results of carbapenem-hydrolysis assays.

## Conclusions

The main limitation of this study is the fact that we were not able to determine its source. Since the outbreak had subsided by applying routine CPE prevention practices, we believe that regardless of its initial source, its continued dissemination was limited to our institution. Although it included only colonized patients, this outbreak still required significant institutional attention and resources. More studies are required in order to elucidate the epidemiology of IMI-ECC and the possible role of environmental sources in it's dissemination.

## Data Availability

The datasets used and/or analysed during the current study are available from the corresponding author on reasonable request.

## Abbreviations

CPE: Carbapenemase-producing enterobacterales
ECC: *Enterobacter cloacae* Complex
TASMC: Tel-Aviv Sourasky medical center
ADL: Activities of daily living score
CCS: Charlson clinical score
WGS: Whole genome sequencing
ST: Sequence-type
IMI-El: IMI-producing *E. ludwigii*
MDR: Multi-drug resistant
ANI: Average nucleotide identity
